# Recommendations for embedding simulation in health services

**DOI:** 10.1186/s41077-023-00262-3

**Published:** 2023-10-06

**Authors:** Ellen Davies, Adam Montagu, Victoria Brazil

**Affiliations:** 1 Adelaide Health Simulation, Faculty of Health and Medical Sciences, The University of Adelaide, Adelaide, SA Australia; 2 Translational Simulation Collaborative, Faculty of Health Sciences and Medicine, Bond University, Gold Coast, QLD Australia; 3 Gold Coast Health Simulation Service, Gold Coast Hospital and Health Service, Gold Coast, QLD Australia

**Keywords:** Simulation consulting service, Healthcare simulation, Health network, Recommendations, Implementation, Tertiary healthcare, Translational simulation

## Abstract

Aspirations to achieve quality and safety goals in health services through simulation have led to significant investments in simulation equipment, space and faculty. However, the optimal governance and operational models through which these resources are expertly applied in health services are not known. There is growing evidence supporting ‘service’ models for simulation. In these models, simulation activities are co-designed and delivered by a team of simulation experts in partnership with health service units, specifically targeting quality and safety goals. Embedded simulation specialist teams working within these programs offer benefits not fully captured by traditional models of health education or by traditional systems for quality and safety.

In this article, we explore broad and specific recommendations for establishing a simulation consultancy service within an Australian metropolitan health service. We base these recommendations on a review of current Australian practice and healthcare simulation literature, and on a specific example within a large outer metropolitan health service. The broad domains discussed include (1) governance and leadership; (2) human resources; (3) principles and planning; (4) operationalise and evaluate and (5) look to the future.

The recommendations recognise that healthcare simulation is moving beyond solely addressing individual learning outcomes. The value of simulation addressing organisation and system objectives through various simulation modalities is increasingly being explored and demonstrating value. There is a growing demand for translational simulation in these contexts, and a consequent requirement for organisations to consider how simulation services can be successfully operationalised. Recommendations included in this paper are discussed and described with the intent of facilitating a deeper appreciation of the complexities associated with, and opportunities afforded by, a well-integrated simulation service.

## Introduction

Evidence supporting the benefits of simulation activities in hospitals and health service environments is substantial. Healthcare simulation has demonstrated a positive impact on individual and team performance [1, 2]. It has been used to implement system responses to quality and safety concerns, enhance technical and behavioural skills, test new practices and pathways of care, and provide clinical education and training [2–4]. As the evidence becomes more compelling, health networks and services across the globe are investing in facilities and equipment, as well as in dedicated simulation personnel to coordinate and liaise with internal stakeholders, to respond to local and organisation-wide concerns, and to evaluate the impact of simulation activities on staff and patient outcomes [2].

However, simulation programs can fail to realise their potential to support quality and safety outcomes for health services. Inconsistent and ad hoc approaches to resourcing, staffing and operational delivery can leave simulation programs struggling to demonstrate a return on investment. Even when simulation activities have been successfully implemented in some segments of a health service organisation, issues of broader distribution and adoption can be problematic, and lead to ‘simulation silos’ [5]. The paradigm in which healthcare simulation operates in a health service may also be a limiting factor. Many health services hold a traditional view of simulation—as an education and training technique. An explicit shift toward simulation that is directly focused on quality and safety outcomes—translational simulation—through exploring and testing system performance is likely to improve outcomes.

While many Australian health services have simulation programs, no formal surveys or assessment of the extent of these programs has been studied or published. Our understanding of the simulation landscape in Australian health services is shaped by our collaborations, partnerships, and discussions with health service leaders and simulation community members. Based on these collective experiences and this expertise with health simulation in Australia, our synopsis is that industry-leading simulation programs in Australia are (1) embedded within the health service; (2) aligned with the Quality and Safety governance of their organisations; (3) comprised of a team of dedicated, expert simulation staff; (4) operating within a ‘service’ model; (5) providers of faculty development to increase organisational capacity to deliver simulation and (6) adopting a programmatic approach to simulation, rather than discrete simulation activities.

In this paper, we describe recommendations provided to an Australian metropolitan health service for establishing a Simulation Consultancy Service. Recommendations were crafted based on a thorough review of the organisation’s existing simulation activities, available facilities and equipment and staff experiences and attitudes towards simulation. They are informed by the experience and expertise of the authors, and the current evidence relating to simulation activities in tertiary hospital settings. These overarching areas of focus may be relevant and useful to others who are preparing and advocating for the organisation, coordination and improvement of health simulation services.

## Main text

### Context

The authors of this paper include the Director and Research Program Lead from Adelaide Health Simulation (AHS) (AM and ED), and the Medical Director of the Gold Coast Health Simulation Service (VB). In 2022, we accepted a contract from the Northern Adelaide Local Health Network (NALHN) executive committee, to undertake a review of existing simulation activities within their health services, and to provide recommendations that would progress the organisation’s agenda of establishing a coordinated and functional simulation program.

NALHN is one of five state government funded health services in South Australia. It supports the health needs of 32% of the metropolitan population of South Australia, and employs just over 6000 staff to deliver healthcare in critical care, acute care and primary health care services [6]. Its services include two hospitals: the Lyell McEwin Hospital and Modbury Hospital; inpatient and community mental health services; the Watto Purruanna Aboriginal Primary Health Care Service and; primary health, sub-acute and transitional care services via a number of GP Plus healthcare clinics.

When the report was written, the expertise of two experienced simulation technicians from AHS informed the findings relating to the quantity and quality of existing simulation equipment and facilities.

### Process for developing recommendations

The strategy, governance and operational models for a successful simulation program must match the institutional context. The overarching health service scope, mission and values will shape how simulation can best serve that mission, as will the governance structures and funding models. The prior experience and current resourcing of simulation within the health service will also be relevant. As a result of these factors, our first step in developing recommendations was to understand the context of NALHN’s health services.

Our work for NAHLN provides an example of how evidence and experience can inform recommendations that are shaped for a particular context. We collected and analysed data from organisation-wide surveys, interviews with key stakeholders and a detailed audit. Interviews were undertaken with a broad array of clinicians, representing various health professions (nursing, medicine, midwifery, allied health) from across the major disciplines (emergency, intensive care, anaesthetics, medical and nursing education, surgery, obstetrics and gynaecology). Questions explored in these interviews related to the current state of simulation activities within the organisation, experiences, perceptions, attitudes towards the various simulation modalities, and aspirations for future engagement with simulation activities or services. Data were analysed thematically, with findings largely constructed within a ‘SWOT’ (strengths, weaknesses, opportunities, threats) framework [7].

Three surveys were administered online—one for clinicians, one for educators and one for members of the organisation’s executive team and divisional directors. Surveys included demographic questions, and modified questions from the barriers survey proposed by Salvoldelli [8, 9]. A descriptive statistical analysis of survey results was delivered to the organisation and provided additional insights into the attitudes, experiences and perceptions of these three cohorts of stakeholders.

Data from these collection points allowed us to have a deeper appreciation of the varied attitudes and perceptions of staff towards health simulation modalities; an understanding of perceived barriers and facilitators to establishing a coordinated simulation program and some ideas for how the organisation might consider implementing a program of simulation. Findings from interviews and surveys were considered in relation to the broad evidence base of academic literature and health simulation principles. We drafted recommendations for the organisation based on current evidence implementing health simulation into health services, applied to the specific context we had explored.

### Recommendations

**Fig. 1:**
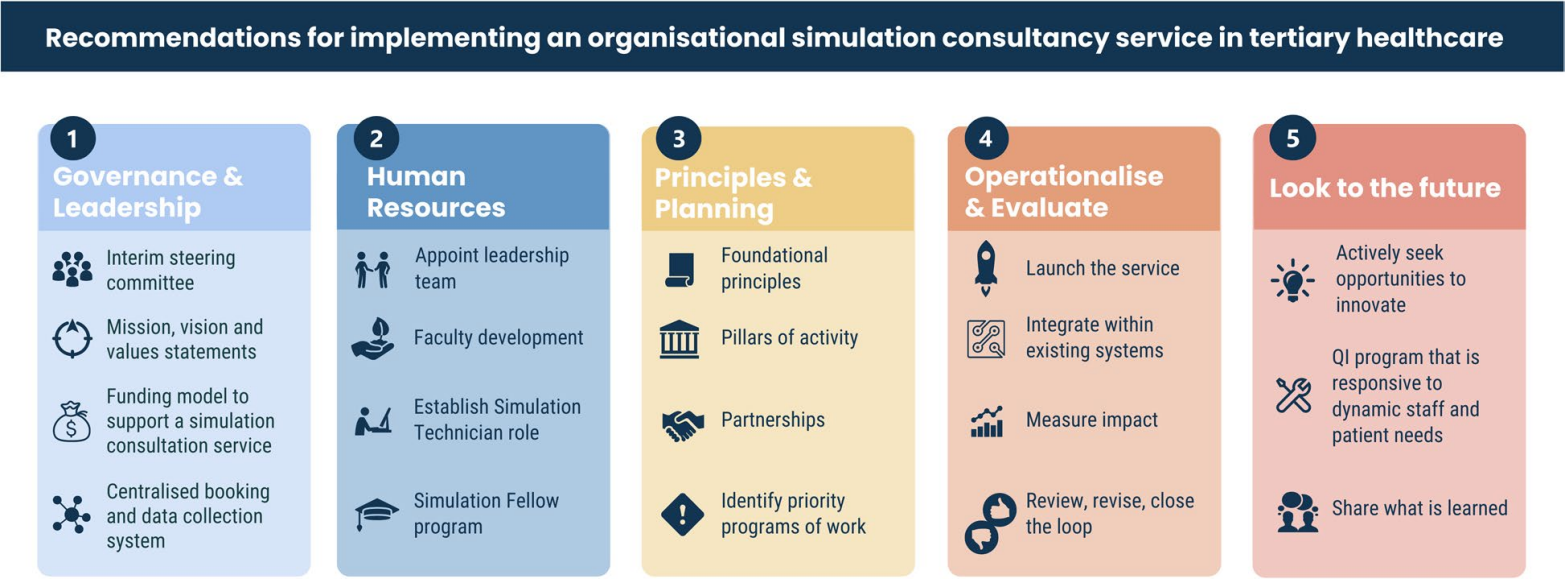
Overview of recommendations

Nineteen recommendations were made, under five broad domains: (1) governance and leadership; (2) human resources; (3) principles and planning: (4) operationalise and evaluate and; (5) look to the future (Fig. 1). The recommendations are not presented as a linear pathway to the design and development of a simulation service. Rather, they are interconnected domains, with various degrees of inter-dependence between many of the recommendations. These interconnections and relevant institutional context will impact on the timing, quality and capacity of a Simulation Consultancy Service to deliver a simulation program within an organisation or health service. In this section we provide recommendations for each domain in summary tables, supported by brief explanatory comments.

#### Governance and leadership

Determining who will lead a Simulation Consultancy Service, how it will be operationalised, and to which part of the organisation they will report, is a key priority. The first recommendation in the domain ‘Governance and Leadership’, is that the organisation establish or work with an established steering committee of inter-professional and inter-disciplinary stakeholders who can review findings regarding context, consider recommendations, and begin the preliminary task of advising on the direction and composition of the Simulation Consultancy Service. Given the opportunities for health simulation to improve patient outcomes and patient safety, and prior success in other similarly sized organisations, our strong recommendation is that the Simulation Consultancy Service should report to, and be aligned with, the Patient Quality and Safety division of the organisation. This group can report to the Executive Committee of the organisation and advise on (1) the appointment of a leadership team for the Simulation Consultancy Service; (2) the funding requirements to support a Simulation Consultancy Service in the short-, medium- and long-term; (3) initial investments required for infrastructure, (for example session booking and data collection systems).

A broadly recommended activity for simulation services, and functional units more broadly, is that vision, mission and values statements are devised to guide establishment, strategic planning, activities and program evaluation [7, 10, 11]. These statements are integral to the standards set by major global simulation societies, including the Society for Simulation in Healthcare [12]. We recommend that a process that engages relevant stakeholders and champions of simulation within the organisation are consulted in this process, so that these statements feel relevant to the organisational context and can be used to direct the Simulation Consultancy Service as it is established.

#### Human resources

Key to progressive and established health service simulation programs is the employment of a team of staff in substantive simulation-specific roles. This team often works to build the capacity of the staff within the organisation to increase overall capacity and to develop and deliver simulation activities [13]. High-performing simulation teams may include program leaders, simulation coordinators, simulation technical experts and clinician content experts. Established programs may include simulation fellows or trainees. Examples of these roles and responsibilities are outlined in Table 1.

**Table 1:** Domain 1—Governance and leadership

Domain	Sub-domain	Recommendations
Governance and leadership	Initial steering committee	Establish, or work with an established, inter-professional, inter-disciplinary simulation steering committee
		Task committee with liaising with the executive committee, simulation champions, and organisation more broadly to guide implementation of the proposed Simulation Consultancy Service
	Mission, vision and values statements	Devise mission, vision, and values statements to guide the Simulation Consultancy Service
		Use these statements to guide decisions made by, and with the Simulation Consultancy Service
	Funding model to support a Simulation Consultancy Service	Invest in people who can progress an agenda of building a Simulation Consultancy Service as outlined in ‘Human Resources’
		Structure funding model to appropriately recognise the requirement of simulation equipment and facilities to be procured, maintained, repaired, and periodically replaced
	Centralised booking and data collection system	Invest in an electronic database for: - Archiving scenario documents (e.g. set-up sheets) - Recording simulation events and activities - Recording attendance and participant feedback
		Invest in an electronic booking system for - Centrally booking simulation facilities - Centrally booking simulation equipment

The size of a Simulation Consultation Service will be determined by factors such as the size of the organisation, the resources available to fund the employment of dedicated staff, and the model of service delivery that is adopted. Beyond those employed directly within a Simulation Consultancy Service, there is also a significant opportunity to support staff throughout an organisation to design and deliver simulation activities, and to co-design programs of simulation activities, as exemplified in other organisations [11, 14, 15]. Table 2 details four sub-domains of recommendations relating to human resources, including (1) a Simulation Service leadership team; (2) a faculty development program; (3) simulation technician role(s) and (4) a Simulation Fellow program.

An active simulation faculty development program can build capacity for simulation delivery, through enhanced skills in design, delivery and debriefing, and through building a simulation community of practice within the organisation [13, 16]. Faculty development may include structured workshops, informal mentoring and support for longitudinal learning pathways, and may also involve partnerships with educational and academic organisations.

For simulation directly focused on quality and safety goals, practitioners may need additional knowledge and skills that build on those required for educationally focused simulations, drawing upon expertise from fields such as safety science, quality improvement, and change management. Developing relationships with experts in these fields to address and explore safety goals will involve networking within the health service organisation or building partnerships externally. An example of simulation experts partnering with clinicians and with quality improvement experts is illustrated in a study at Gold Coast University Hospital in which significant improvements in care of women suffering post-partum haemorrhage was achieved [15].

**Table 2:** Domain 2—Human resources

Domain	Sub-domain	Recommendations
Human resources	Leadership team for Simulation Consultancy Service	Appoint a leadership team for the Simulation Consultancy Service
		Task the leadership team with establishing the unit to align with the developed mission, vision, and values statements and best-practice evidence available from academic literature
	Faculty development	Up-skill faculty who already coordinate simulation activities, and those with clinical education portfolios
		Build a shared mental model and understanding of simulation modalities across the organisation
	Establish Simulation Technician role	Employ at least one simulation technician at each major site to manage and maintain the organisation’s portfolio of simulation equipment
		Task the simulation technician with advising on current and future simulation facility and equipment procurement, maintenance and replacement
	Simulation Fellow program	Establish a Simulation Fellow Program to train clinical staff, in a placement model, to lead simulation activities and work with the various teams in the organisation

#### Principles and planning

Simulation delivered in a health setting needs to be viewed and implemented as an organisational strategy, and not a discrete event or series of unconnected events [4, 17]. The four sub-domains of recommendations presented in Table 4 are significantly inter-related. They emphasise the considerations required to develop a program of simulation that is relevant and acceptable to people throughout the organisation and that is coherent and responsive to the needs of the organisation.

Firstly, in developing an organisational strategy for designing and delivering simulation activities, we recommend that the underlying principles that will guide this strategy are defined. Examples of foundational principles may include the following:

1. Simulation activities are linked to the medium and long-term organisational strategies for improving the quality and safety of service provision. Simulation activities are linked to health quality and safety standards, for example:
  - National Safety and Quality Health Standards (Australia) (https://www.safetyandquality.gov.au/standards/nsqhs-standards)
  - Canadian Quality and Patient Safety Framework for Health Services (Canada) (https://www.healthcareexcellence.ca/en/resources/canadian-quality-and-patient-safety-framework-for-health-services/)
  - NHS Patient Safety Strategy (England) (https://www.england.nhs.uk/patient-safety/the-nhs-patient-safety-strategy/#patient-safety-strategy)
2. Safety-I and Safety-II principles are considered when designing simulation activities and the debriefing points for these activities. That is, consideration is given to how the service can use simulation modalities to ensure that:
  - As little as possible can go wrong (Safety-I), and
  - As much as possible can go right (Safety-II) [18]
3. Psychological safety principles for simulation are incorporated at all stages of development and delivery of simulation activities.
4. Simulation activities and resources are accessible across the service, inclusive of all disciplines, professions and teams.

Secondly, the stated principles should then underpin the internal structure and function of the Simulation Consultancy Service, i.e. ‘pillars’ of activity may include domains such as ‘translational simulation’, ‘education and training’, ‘faculty development’, ‘simulation innovation’, ‘guideline testing’ and ‘scholarship and research’. Examples of pillars of activity can be seen in the Boston Children’s Hospital, who have named three pillars: “T*raining and Performance: Accelerating clinical training and high performance*”; “*Human Factors and Systems Design: Engineering out hazards and improving safety in patient care*” and; “*Device Design Solutions: Just-in-time innovation for health care*” [19]. Examples of other standards and considerations when formulating foundational principles and pillars of activity for a Simulation Program are available in several recent publications including Baxendale et al.’s [20] scoping review that reports on standards for in-situ simulation, Brock et al.’s [21] description of a simulation program for Paediatric Critical Care Fellows and, the ‘Input-process-output’ framework for translational simulation published in 2021 [17].

The third recommendation in the ‘Principles and Planning’ domain, relates to partnerships (Table 3). Healthcare simulation is deeply rooted in partnerships. Partnerships between novices and experts; clinicians and non-clinicians; people from different disciplines and professions; institutional leaders and the people who lead simulation teams; simulated patients, simulation technicians, simulation coordinators and learners. The recommendation we make here may sound simplistic, but it is through partnerships that great opportunities for innovation, learning, and excellence in the delivery of healthcare simulation and healthcare delivery flourish.

**Table 3:** Examples of roles and role descriptions

Role	Responsibilities	Desired characteristics
Clinical director/program leader	• To lead a new Simulation Consultancy Service in its mission to optimise healthcare service delivery • To co-design and develop a strategic plan for simulation activities that is responsive to current and future needs • To enhance healthcare delivery through the implementation of simulation programs that promote excellence, innovation, skill development and team performance • To partner with departments and units across the organisation to develop solutions to clinical concerns and problems • To coordinate faculty development across the organisation with the intent of building a shared mental model and understanding of simulation modalities and opportunities • To be accountable within the organisation for the financial management of the service, simulation resources and facilities	• Previous leadership positions with responsibilities for people and finances • Demonstrated experience in developing and delivering high-quality simulation activities • Evidence of positive working relationships within a health simulation community • Evidence of scholarship and research outputs related to health simulation • Ability to work in a team and manage professional relationships effectively
Simulation coordinators	• Active involvement in the development and delivery of simulation activities • Coordination and consultation with departments and units across the organisation to co-design and develop a program of simulation activities that provides appropriate professional development and progresses an agenda of excellence in healthcare service delivery • To assist with coordinating faculty development across the organisation with the intent of building a shared mental model and understanding of simulation modalities and opportunities • Advise on simulation resources and facilities	• Demonstrated experience in developing and delivering simulation activities • Demonstrated experience in delivery of innovative and effective education and curriculum development • Ability to work in a team and manage professional relationships effectively
Simulation technician	• Assist with installation testing, operation and maintenance activities of simulation equipment and simulation related IT network systems • Ensure simulation facilities are appropriately set up for simulation activities • Maintain appropriate stock levels • Assist in the delivery of teaching and set-up of complex equipment, including hi-fidelity manikins • Assist in ensuring simulation activities adhere to Work Health and Safety regulations • Provide technical support to Simulation Coordinators and the Clinical Director	• Working understanding of IT networks and systems • Experience with simulation equipment and its maintenance • Demonstrated knowledge of stock control and management using a database • Demonstrated experience in managing multiple tasks with competing deadlines • Ability to work in a team and manage professional relationships effectively
Simulation fellow	• Perform in the role of a simulation coordinator for a time-limited period • Achieve tailored learning objectives related to the development and delivery of health simulation	• Enthusiastic about learning and implementing the modalities of healthcare simulation • Ability to work in a team and manage professional relationships effectively

For example, when the COVID-19 pandemic posed challenges for maternity services at the Gold Coast University Hospital, the simulation service was able to partner with their team to undertake diagnostic simulations that tested new processes, and identified logistical, communication and coordination issues [14]. Through pursing a translational simulation process, this partnership between the simulation consultancy unit and maternity services resulted in refined processes and ultimately improved patient and staff safety [14].

The three categories of people we have included in these recommendations are the individuals within the organisation who have simulation expertise (not employed within the simulation service, but in the service more generally); teams, units and professions within the service and experts beyond the organisation (Table 4). This may be expanded to also include end-users (patients, family members) and other health service stakeholders.

**Table 4:** Domain 3—Principles and planning

Domain	Sub-domain	Recommendations
Principles and planning	Foundational principles	Define the foundational principles that will guide simulation activities—these should align with best available evidence and with the vision, mission and values statements that have been developed
	Pillars of activity–internal structure of the service	Define the pillars of activity that will be coordinated by the Simulation Consultancy Service—these should align with best available evidence, the vision, mission and values statements and the unit’s foundational principles
	Partnerships	Foster partnerships with individuals within the organisation who have simulation expertise and who will champion simulation activities amongst their peers
		Partner with teams, units and professions throughout the organisation to develop simulation activities that meet their needs
		Partner with experts beyond the organisation to build and share capacity and expertise
	Identify priority programs of work	Work with the quality and safety unit, clinical educators, the executive team, clinical units and other teams to identify areas of clinical, technical and performance concern
		Devise a plan for prioritising simulation activities throughout the organisation

Finally, the fourth recommendation in this domain is ‘Identifying priority areas of work’. As noted in recent literature, buy-in from participating units is necessary for session objectives to be met [11, 15, 22]. Pathways to identifying priority areas of work may be found through (1) identifying the individuals and units who are enthusiastically willing to participate in simulation activities and (2) identifying challenges faced throughout the organisation that are amenable to change or improvement through the design and delivery of simulation activities. As an exemplar, Trawber, Sweetman (5) successfully implemented a process that facilitated the identification of both the simulation enthusiasts and situations in which simulation activities could be implemented at the Fiona Stanley Hospital in Western Australia. The ‘Simulation to Enhance Patient Safety (STEPS) Referral Pathway’ provided a streamlined mechanism for translational simulation to be requested, prioritised and planned [5].

#### Operationalise and evaluate

Implementation of any simulation program strategy presents challenges in overcoming anticipated and unanticipated barriers [23]. Principles drawn from knowledge translation and implementation science literature that can assist with increasing the likelihood of success [24, 25]. The recommendations presented in Table 5 are not exhaustive, but may enhance acceptability and engagement.

**Table 5:** Domain 4—Operationalise and evaluate

Domain	Sub-domain	Recommendations
Operationalise and evaluate	Launch the service	Formally launch the Simulation Consultancy Service with clear, organisation-wide communication about purpose, communication pathways, pillars of activity and initial priorities
		Launch a single point of contact for staff to request simulation consultancy services
	Integrate within existing systems	Work with existing units, teams, services, and groups to integrate a program of simulation activities
		Demonstrate connectivity with existing systems
	Measure impact	Build capacity to measure impact of the Simulation Consultancy Service, discrete simulation events and the emerging simulation program on, for example, service delivery, patient safety, staff satisfaction, staff and team performance
	Review, revise, close the loop	Develop formal mechanisms for regularly and routinely reviewing the implementation of the Simulation Consultancy Service
		Evaluate the impact of simulation activities that aimed to address a specific organisational need, concern or problem, and formally report these outcomes

Evaluating the impact and effectiveness of a simulation consultancy service is not a straightforward endeavour [26, 27]. Methods to evaluate effectiveness and impact have included self-report, observation, and calculation of quantitative cost effectiveness [28]. However these strategies have often drawn upon an educational evaluation paradigm (e.g. Kirkpatrick’s 4 levels of evaluation) [29]. By contrast, simulation programs that are funded and embedded within health services should demonstrate return on investment (ROI) that is relevant to patients and health services [28, 30]. Drawing upon quality improvement frameworks may be more appropriate, for example, the quadruple aims of reducing costs; improving population health, patient experience, and team well-being [31]. While a granular discussion of ROI is beyond the scope of our recommendations, we strongly suggest evaluations strategies are developed contemporaneously with the program mission, vision and scope [25].

#### Look to the future

Healthcare simulation is an evolving practice, with opportunities to innovate and proactively respond to the dynamic needs of patients, families, staff, and the broader community. A significant role for any Simulation Consultancy Service is the careful consideration of which opportunities will be impactful, how and when to implement new techniques, technologies and programs, and who to partner with to accomplish desired outcomes (Table 6).

**Table 6:** Domain 5—Look to the future

Domain	Sub-domain	Recommendations
Look to the future	Actively seek opportunities to innovate	Work with internal stakeholders to discover what simulation activities are effective and what activities could be enhanced to improve outcomes
		Connect with industry leaders and innovators to understand what is working in other areas, and how this could be applied to the local context
	QI program that is responsive to dynamic staff and patient needs	Be aware of dynamic system needs and match appropriate simulation modalities to meet these needs
		Work with Quality and Safety teams to discover threats to patient and staff safety and implement appropriate simulation events to reduce these threats
	Share what is learned	Generously share what is learned in this process of designing and implementing a new Simulation Consultancy Service with internal and external stakeholders
		Share what is learned from delivering simulation activities. This sharing of information may be in the form of: i) Participating in local, national and international forums, seminars and conferences ii) Attending or initiating a community of practice iii) Publishing findings in academic journals

In *looking to the future,* we encourage and recommend new and established simulation units actively seek opportunities to be innovative, to be responsive to the dynamic needs of patients and staff, to formally evaluate their impact and to generously share what is learned in these processes with the broader simulation, research and health professional communities.

## Discussion

In formulating and presenting the recommendations outlined above, there is tension between presenting overly prescriptive guidelines that are not relevant across contexts, and overly generic advice with inadequate detail to be helpful. Recognising this tension, we have provided broad principles as well as detailed recommendations that we hope will facilitate a successful implementation journey. Three underlying principles were considered in the development of these recommendations. These include (1) an operational model of ‘consultancy service’, (2) a focus on people, and not equipment and facilities, and (3) a deep appreciation of context.

### Consultancy service model

It is our strong recommendation that an empowering, support-focused ‘service’ model be adopted for simulation programs operating within health services. This model situates simulation as a core function in the health service and recognises that many health service staff may be involved in simulation design and delivery. The role of the Simulation Consultancy Service is thus positioned to support those staff with expertise in scenario design, technical delivery, equipment and leading or supporting learning conversations. This model recognises that those needs will vary enormously between departments or units within the health service and has seen success in other Australian health networks.

### Focus on people, not just equipment and facilities

Significant government and organisational capital investment have been poured into simulation equipment and facilities over the past two decades [32, 33]. Despite these capital investments, coordinated and effective simulation programs have rarely materialised without a serious co-investment in people and faculty development. As argued by Lazzara, Benishek [4], the trend towards leaner health workforces has limited the opportunity for dedicated simulation staff to be employed to lead and work in such programs, to the detriment of these organisations. A lack of dedicated staffing is particularly problematic when considering that health professions’ training within health services should be considered in the context of a system, and not as discrete learning events [34, 35]. As described in our recommendations, this involves adequate resourcing for dedicated simulation staff with complementary skill sets, faculty development programs, and upskilling for educators and quality improvement staff in using simulation.

### Contextual relevancy

The successful implementation of new or altered processes and structures requires an understanding of the context where this change will occur [23]. The recommendations described here may not all be relevant to all contexts. Hence, we recommend an exploratory process should be undertaken to deeply understand the institutional context, prior to planning a simulation consultancy service. This may be undertaken internally, or (as in our experience) by an external expert group. It is the people, physical and geographical environments and culture of organisations that will both inform and impact the success of the service.

## Conclusion

With growing interest in organisation-wide health simulation programs, there are exciting opportunities to thoughtfully design models for service delivery that are progressive, sustainable, guided by evidence, and responsive to the dynamic and complex nature of health organisations. In this article, we offer several recommendations for designing and launching a simulation consultancy service that will support the quality and safety goals of a contemporary health service, whether multi- or single site. This design process has been guided by theoretical lenses: relevant principles from the implementation science and change management literature. The design has also been informed by published examples of simulation service configurations and activities, and through the sharing of knowledge and experiences between services. We anticipate and encourage discussion and debate amongst our health education and simulation colleagues relating to this evolving landscape and look forward to seeing how health services progress to incorporate cohesive simulation services, sophisticated enough to lead to positive impact and outcomes.

## Data Availability

Not applicable.

## Abbreviations

AHS: Adelaide Health Simulation
NALHN: Northern Adelaide Local Health Network
ROI: Return on Investment
STEPS: Simulation To Enhance Patient Safety
SWOT: Strengths, Weaknesses, Opportunities, Threats

## References

[1] Owen H (2016). Simulation in healthcare education.

[2] Brazil V, Purdy E, Bajaj K (2023). Simulation as an improvement technique.

[3] Minors AM, Yusaf TC, Bentley SK, Grueso D, Campbell-Taylor K, Harford M (2023). Enhancing safety of a system-wide in situ simulation program using no-go considerations. Simul Healthc..

[4] Lazzara EH, Benishek LE, Dietz AS, Salas E, Adriansen DJ (2014). Eight critical factors in creating and implementing a successful simulation program. Jt Comm J Qual Patient Saf.

[5] Trawber RAH, Sweetman GM, Proctor LR (2021). Improving simulation accessibility in a hospital setting: implementing a simulation consultation service. Simul Healthc.

[6] Government of South Australia. Northern Adelaide Local Health Network Strategic Plan 2020–2025. 2020. Available from: https://www.sahealth.sa.gov.au/wps/wcm/connect/public+content/sa+health+internet/resources/nalhn+strategic+plan+2020-25.

[7] O’Connor P, O’Dowd E, Lydon S, Byrne D. Developing a strategic plan for a healthcare simulation facility. Int J Healthc Simul. 2023.

[8] Decarlo D, Collingridge DS, Grant C, Ventre KM (2008). Factors influencing nurses’ attitudes toward simulation-based education. Simul Healthc.

[9] Salvoldelli GL, Naik VN, Hamstra SJ, Morgan PJ (2005). Barriers to use of simulation-based education. General Anaesth.

[10] Appelbaum SH, Habashy S, Malo JL, Shafiq H (2012). Back to the future: revisiting Kotter’s 1996 change model. J Manag Dev.

[11] Schmutz JB (2022). Institutionalizing an interprofessional simulation education program: an organizational case study using a model of strategic change. J Interprof Care.

[12] Society for Simulation in Healthcare. Society for simulation in healthcare accreditation. Core Accreditation Standards2021.

[13] Peterson DT, Watts PI, Epps CA, White ML (2017). Simulation faculty development: a tiered approach. Simul Healthc.

[14] Lowe B, De Araujo V, Haughton H, Schweitzer J, Brazil V (2020). Preparing maternity for COVID-19: a translational simulation approach. Aust N Z J Obstet Gynaecol.

[15] Brazil V, McLean D, Lowe B, Kordich L, Cullen D, De Araujo V (2022). A relational approach to improving interprofessional teamwork in post-partum haemorrhage (PPH). BMC Health Serv Res.

[16] Paige J, Graham L, Sittner B (2020). Formal training efforts to develop simulation educators: an integrative review. Simul Healthc.

[17] Nickson CP, Petrosoniak A, Barwick S, Brazil V (2021). Translational simulation: from description to action. Adv Simul (Lond).

[18] Hollnagel E (2014). Safety-I and safety-II : the past and future of safety management.

[19] Boston Children’s Hospital. Immersive design systems. Available from: https://www.childrenshospital.org/clinician-resources/immersive-design-systems-landing-page.

[20] Baxendale B, Evans K, Cowley A, Bramley L, Miles G, Ross A (2022). GENESISS 1-Generating Standards for In-Situ Simulation project: a scoping review and conceptual model. BMC Med Educ.

[21] Brock KE, Tracewski M, Allen KE, Klick J, Petrillo T, Hebbar KB (2019). Simulation-based palliative care communication for pediatric critical care fellows. Am J Hosp Palliat Care.

[22] Eller S, Rudolph J, Barwick S, Janssens S, Bajaj K (2023). Leading change in practice: how “longitudinal prebriefing” nurtures and sustains in situ simulation programs. Adv Simul (Lond).

[23] Harvey G (2022). Context matters, so how do we get better at working with context in implementation research and practice? Comment on “stakeholder perspectives of attributes and features of context relevant to knowledge translation in health settings: a multi-country analysis”. Int J Health Policy Manag.

[24] Lane-Fall MB, Curran GM, Beidas RS (2019). Scoping implementation science for the beginner: locating yourself on the “subway line” of translational research. BMC Med Res Methodol.

[25] Wensing M, Grol R (2019). Knowledge translation in health: how implementation science could contribute more. BMC Med.

[26] Abildgren L, Lebahn-Hadidi M, Mogensen CB, Toft P, Nielsen AB, Frandsen TF (2022). The effectiveness of improving healthcare teams’ human factor skills using simulation-based training: a systematic review. Adv Simul (Lond).

[27] Lin Y, Cheng A, Hecker K, Grant V, Currie GR (2018). Implementing economic evaluation in simulation-based medical education: challenges and opportunities. Med Educ.

[28] Bukhari H, Adreatta P, Goldiez B, Rabelo L (2017). A framework for determining the return on investment of simulation-based training in health care. J Health Care Organ Provision Financ.

[29] Eppich W, Reedy G (2022). Advancing healthcare simulation research: innovations in theory, methodology, and method. Adv Simul (Lond).

[30] Nestel D, Brazil V, Hay M (2018). You can’t put a value on that… Or can you? Economic evaluation in simulation-based medical education. Med Educ.

[31] Bodenheimer T, Sinsky C (2014). From triple to quadruple aim: care of the patient requires care of the provider. Ann Fam Med.

[32] Nestel D, Bearman M, Brooks P, Campher D, Freeman K, Greenhill J (2016). A national training program for simulation educators and technicians: evaluation strategy and outcomes. BMC Med Educ.

[33] Rudd C (2013). Enhancing the uptake of learning through simulation in health.

[34] Salas E, Tannenbaum SI, Kraiger K, Smith-Jentsch KA (2012). The science of training and development in organizations: what matters in practice. Psychol Sci Public Interest.

[35] Paige J, Sonesh S, Garbee D, Bonanno L (2020). Comprehensive healthcare simulation: interprofessional team training and simulation.

